# Reply to Letter to the Editor: I Spy With My Little Clinician's Eye

**DOI:** 10.5435/JAAOSGlobal-D-20-00164

**Published:** 2020-01-12

**Authors:** Rodrigo Guiloff, Andrés Schmidt-Hebbel, Alex Vaisman

**Affiliations:** Santiago, Chile

*The Authors' Reply*: The authors appreciate the comments of Dr. Nizić^1^ in response to the publication of the above reference regarding the use of the clinician's eye (CE) as a valid method for the diagnosis of patella alta (PA) and agree with its high specificity. The authors would like to address the comments stated in his letter to the editor:

(1) Sensitivity and specificity of the CE and the “Reference Line” (RL)^2^: As indicated in our introduction, we agree with Dr. Nizić that none of the methods used for the diagnosis of PA is considered a benchmark; therefore, reporting the sensitivity and specificity of a new reference (as the CE and the RL) might be challenging. However, as stated under the methods section of our study, the Caton-Deschamps (C-D) index has already been used to validate the commonly used modified Insall-Salvati ratio (mIS)^3^ and is the preferred method of the musculoskeletal radiologist (mskR), who was considered our benchmark for comparison. In addition, in a review article of different measurements for patellar height by Phillips et al,^4^ the C-D was found to be the only method to have included knees that were asymptomatic, in an endeavor to establish a normal range of values, and was described (next to the Blackburn-Peel) as the most reliable indirect plain-film radiographic technique. Notably, in Chile, unpublished results from a recent study of our research team showed that the C-D is the most common reference used among Chilean orthopaedic surgeons to diagnose PA (Accordingly, we consider the C-D measured by a msk-R as a valid method for assessing our new reference, the CE). We are pleased that our study has motivated Dr. Nizić to review his data and report the sensitivity and specificity of the RL, which are indeed very high (100% and 93%, respectively). However, although we admire his effort, we must say his results might be biased and therefore should be carefully analyzed. As already mentioned, conducting a validation study of a new reference, such as the CE or the RL, requires a benchmark. In our study, we used an external observer, a trained msk-R, as a benchmark, which showed an excellent intraobserver agreement for the C-D (ICC of 0.93). In the author's letter, because they are not specifying the use of an external observer for the comparison, one has to assume that they are using the same observer's measurement as their benchmark, which is not entirely correct. Dependent observations suppose a methodological bias for the results. This would be more accurately defined as a correlation between different measurements by the same observer, rather than a correct method for establishing sensitivity and specificity. We encourage Dr. Nizić to conduct a new study using the C-D measurements of a validated external observer, such as an experienced msk-R, to assess their RL's sensitivity and specificity. Likewise, he might include the CE as a comparing measurement. Until then, we believe that there is no scientific basis for accounting the sensitivity and specificity of the RL, nor to compare it with the CE.
(2) CE fundament: Dr. Nizić has wondered how the CE works and has questioned what the observers considered while using this method. First, we must state that we do not entirely know the answers to these questions; to do so, one must fully understand how the human vision and brain circuits work, which are not fully understood, and is out of the scope of an orthopaedic article. That being said, we may contribute to the discussion by clarifying that to avoid any possible bias and to permit a fair comparison between observers and evaluate the influence of years of experience in orthopaedic surgery (besides asking the observers to use only their vision, without any physical measurements, to answer if they consider that the image had a PA or not), no instructions were given before applying the CE. Thanks to Dr. Nizić’s conjecturing, we agree with his comment that “our vision is a measuring instrument,” and as such, it may be trained. Distance perception accuracy has shown to increase with training.^5-7^ That may explain why the more experienced observers (more years of orthopaedic knee practice, therefore more knowledge and application of different standard methods for evaluating patellar height) achieved a higher sensitivity in the CE. After considering these observations, it is our belief that the CE works as a screening tool for the diagnosis of PA, which somehow integrates previous knowledge and gives the observer an estimation of the patellar height. Using one's eyes as a screening method is a common practice in orthopaedics: we use them for estimating coronal or sagittal deformities, measuring the Q-angle, joint range of motion, and many others, which helps us to determine which patients are suitable for the use of complementary images or measurements. Furthermore, visual estimations are not just found in our practice. For example, designers and architects use their visual perception for estimating spaces, chefs for estimating quantities, and even in our daily life activities, such as driving a car, we estimate distances and screen the road to decide if it is safe to overtake a car in front of us. We are pleased with Dr. Nizić's proposition of trying to increase the CE's accuracy by teaching and instructing an observer what to see when evaluating a lateral knee X-ray. Because the RL is an easy measurement, which does not need a physical calculation, we agree with Dr. Nizić that it could be mentally projected while using the CE, improving its accuracy. After this line of reasoning, if more simple landmarks are learned before applying the CE, the higher the sensitivity and specificity of the CE will be, especially those that can be mentally drawn by an observer without the need for physical measurements, such as the RL or the Blummensaat line.^8^ We have started an investigation to prove this hypothesis, and we expect to be able to publish it as soon as we obtain concluding results.

Finally, the authors would like to thank again Dr. Nizić for his interest and stimulating comments on our investigation. His queries have immersed us in a constructive deliberation of our study, which we expect has increased the understanding of our results and will increase the accuracy of the CE.
